# ASSESSING REGULATORY STRINGENCY FOR LICENSED ASSISTED LIVING: A MULTIFACETED APPROACH

**DOI:** 10.1093/geroni/igad104.0210

**Published:** 2023-12-21

**Authors:** Lindsey Smith, Jacy Weems, Cassandra Hua, Sarah Dys, Paula Carder, Kali Thomas

**Affiliations:** Oregon Health & Science University, Portland, Oregon, United States; Brown University School of Public Health, Providence, Rhode Island, United States; University of Massachusetts Lowell, Lowell, Massachusetts, United States; Portland State University, Portland, Oregon, United States; Portland State University Institute on Aging, Portland, Oregon, United States; Johns Hopkins University, Baltimore, Maryland, United States

## Abstract

Regulations in long-term care settings aim to ensure quality and accessibility for residents. However, inadequate enforcement can limit their impact on the community. Simply increasing regulations does not always lead to improved care, as it may incentivize facilities to limit admissions. Regulatory stringency encompasses the scope, specificity, and enforcement of regulations. This study examines 10 dimensions of specificity related to medical care regulations in assisted living, focusing on scope and enforcement. We found that regulations related to staffing levels and resident assessments governed almost all assisted living communities, while medication review and administrator certification were the least commonly addressed. The “Residential Care Facilities” license with an “Alzheimer’s Dementia Care Unit” certification in Maine had the highest stringency score, though the “Assisted Living Program” license in the same state had one of the lowest. High regulatory specificity and scope scores were not always associated with high enforcement. For example, the “Assisted Living Center” license in South Dakota covered 100% of the scope’s dimensions and 75% of the provisions contributing to the specificity score, but had no regulatory enforcement for these rules. Findings suggest that a multifaceted approach to assessing regulatory stringency is crucial for policymakers and researchers to understand regulatory approaches across license types and states. Accounting for differences in licensure is also important for a broad view of regulatory stringency.

